# Wearable fitness tracker use in federally qualified health center patients: strategies to improve the health of all of us using digital health devices

**DOI:** 10.1038/s41746-022-00593-x

**Published:** 2022-04-25

**Authors:** Michelle Holko, Tamara R. Litwin, Fatima Munoz, Katrina I. Theisz, Linda Salgin, Nancy Piper Jenks, Beverly W. Holmes, Pamelia Watson-McGee, Eboni Winford, Yashoda Sharma

**Affiliations:** 1 All of Us Research Program, National Institutes of Health, Bethesda, MD USA; 2 San Ysidro Health, San Ysidro, CA USA; 3 Sun River Health, Peekskill, NY USA; 4 Cooperative Health, Columbia, SC USA; 5 Jackson Hinds Comprehensive Health Center, Jackson, MS USA; 6 Cherokee Health Systems, Knoxville, TN USA; 7 Weitzman Institute, Community Health Center, Inc., Middletown, CT USA

**Keywords:** Preclinical research, Society

## Abstract

As the use of connected devices rises, an understanding of how digital health technologies can be used for equitable healthcare across diverse communities is needed. We surveyed 1007 adult patients at six Federally Qualified Health Centers regarding wearable fitness trackers. Findings indicate the majority interest in having fitness trackers. Barriers included cost and lack of information, revealing that broad digital health device adoption requires education, investment, and high-touch methods.

## Introduction

In an increasingly connected world, mobile devices have become ubiquitous^1^. Wearable devices, including fitness trackers (referred to throughout the paper as “wearables” and “fitness trackers” interchangeably), provide nearly continuous information on physical activity, heart rate, and sleep. As use increases, data are increasingly integrated into clinical and research settings. There is emerging evidence that fitness trackers can identify changes in heart rate variability, potentially identifying COVID-19 onset prior to a clinical diagnosis^2^. However, there is a lack of diversity in studies using wearables to study health outcomes^3^. Despite an increase in broadband and smartphone ownership and use across the United States, access to digital health technologies in lower-income households lags behind middle and upper-income households^4^. Improved access to digital infrastructure and devices in diverse communities is needed to avoid the risk of digital technologies becoming another social determinant of health^5^.

One of the core values of the National Institutes of Health’s (NIH’s) *All of Us* Research Program is diversity in all aspects of the program, including participants, consortium members, program staff, and researchers^6^. Diversity of the underlying data from participants is critical for reducing bias in precision medicine research, which aims to discover best clinical practices at the individual, not population, level. The program welcomes participants from all backgrounds and aims to reflect the rich diversity of the United States by enrolling people from communities that are historically underrepresented in biomedical research (UBR), such as racial and ethnic minority groups and those with limited access to medical care^7^. Recognizing the value of digital health technologies for research and health, the program launched Fitbit Bring-Your-Own-Device (BYOD), enabling participants to donate their Fitbit data to the program^8^. However, when *All of Us* Fitbit participant demographics were compared to all program participants, a reduction in diversity in race and socioeconomic status was noted^9^. This study was designed to reach diverse communities served by Federally Qualified Health Centers (FQHCs) to understand the gaps to participation in Fitbit BYOD.

To bridge this knowledge gap, six FQHCs that are also a part of the *All of Us* Consortium conducted a survey to collect patients’ demographic information, interest in having a fitness tracker, and other factors potentially associated with this interest. Descriptive statistics, univariate and multivariate logistic regression, and qualitative assessment of free-text responses were used to analyze the results (see “Methods” for details; see Supplementary Fig. 1 for a map of participating FQHC sites).

**Fig. 1:**
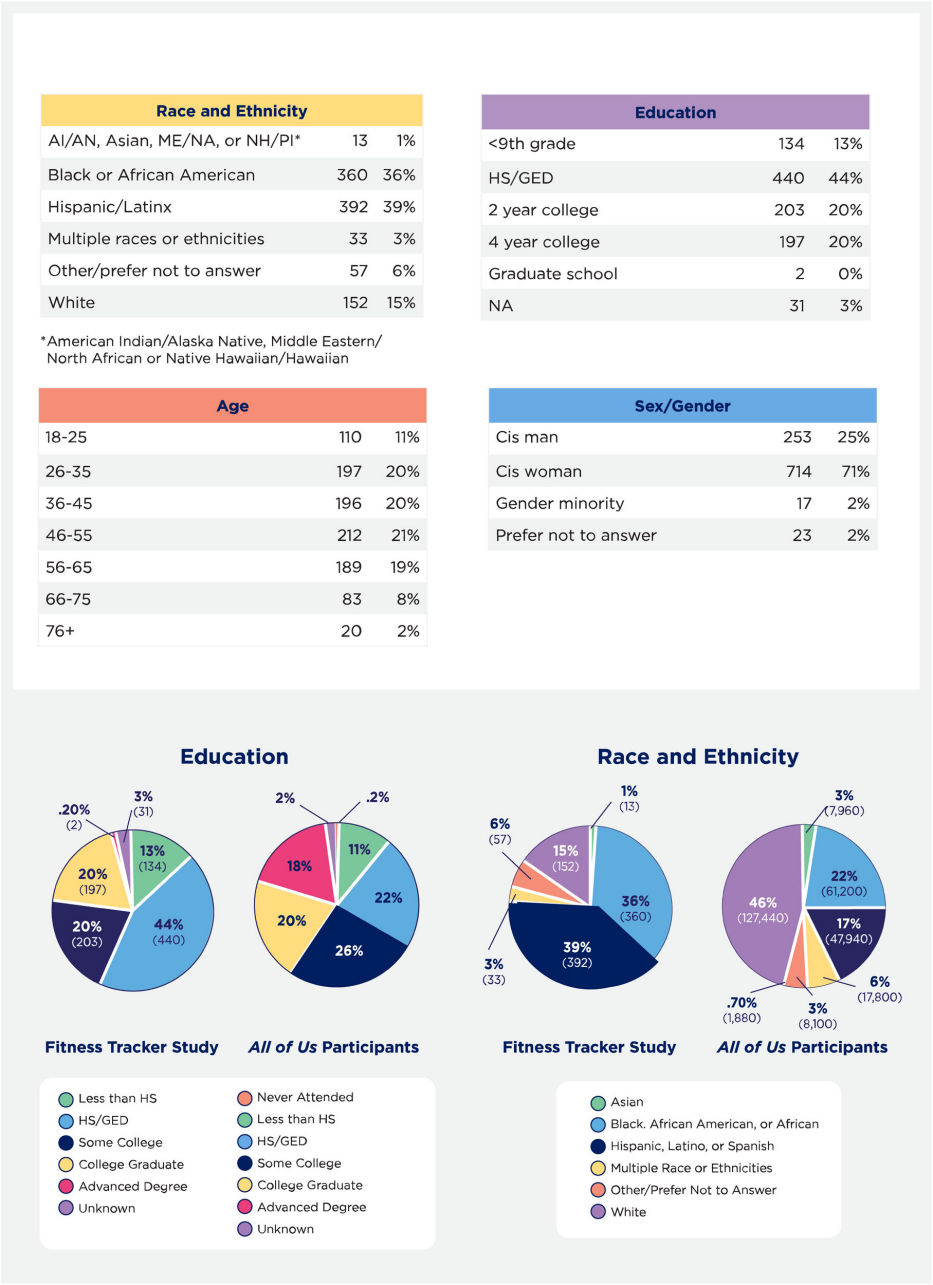
*All of Us* Fitness Tracker Survey demographics. Tables show the race/ethnicity, education, age, and sex/gender characteristics of the survey respondents. Pie charts compare the education levels and race/ethnicity of the survey respondents to *All of Us* Research Program participants. Some *All of Us* participants are FQHC patients and may have taken part in this survey, but not all survey respondents are enrolled in *All of Us*.

Of the 1007 adults surveyed, 39% identified as Hispanic, 36% as non-Hispanic Black or African American, and 15% as non-Hispanic White (Fig. 1). Almost three-quarters identified as cis-gender women (71%), 14% had less than a 9th-grade education while 45% had completed high school, and participants were evenly divided across age groups. The surveys were administered in English (68%) and Spanish (32%). The primary outcome was whether participants would like a fitness tracker, and overall 58% responded “yes,” 20% “no,” and 23% did not answer (Fig. 1).

**Fig. 2:**
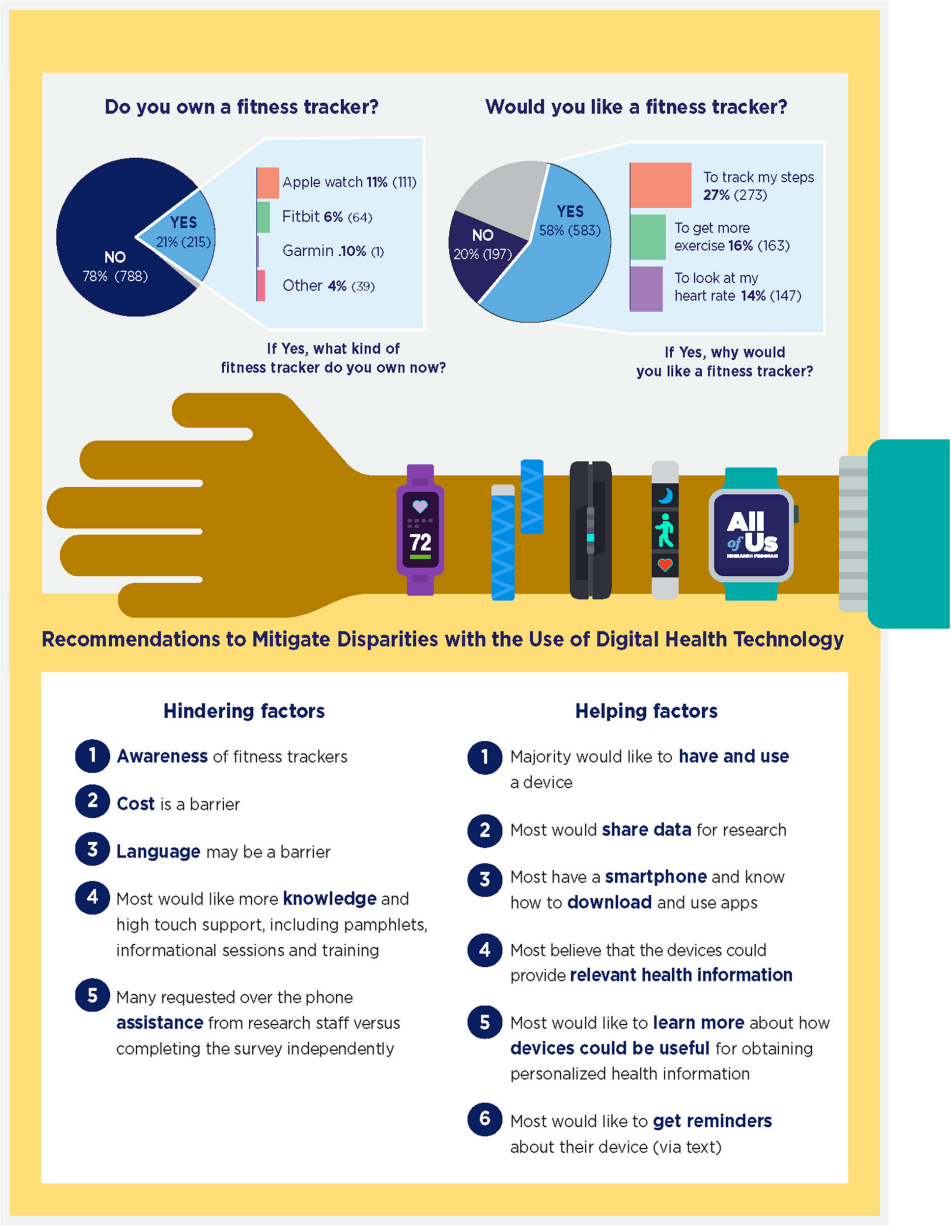
Fitness tracker survey results. Participants were asked a variety of questions centered around their exposure to, ownership of, interest in, and familiarity with fitness trackers. The top two pie charts illustrate participants’ ownership and interest in fitness trackers. Participants were also asked what kinds of things get in the way of owning a fitness tracker. Those items were distilled and are listed under “Hindering factors.” Respondents were also asked about factors that they may consider helpful in reducing barriers to using a fitness tracker, combined under “Helping factors” as recommendations for potential methods to mitigate disparities in digital health technology use.

Participants were asked a variety of questions about their exposure to, ownership of, interest in, and familiarity with fitness trackers. Figure 2 displays participants’ ownership rate and interest in fitness trackers. Participants were asked about barriers to owning a fitness tracker. These “Hindering factors” include cost, a general awareness of fitness trackers, and specific information about how they can provide health insights, language, and assistance over the phone vs fully digital methods. Respondents were also asked about helpful factors for using fitness trackers, combined under “Helping factors” as recommendations for potential methods to mitigate disparities in digital health technology use. These include an interest in having a device and learning about how fitness trackers can be used to track health, a willingness to share data for research, owning a smartphone and knowledge of how to download and use apps, and an interest in learning more.

A number of factors were associated with “would you like a fitness tracker” at the 0.05 significance level using two-sided tests (Table 1). Participants who responded they would like a fitness tracker had higher odds in univariate logistic regression models of identifying as a cis woman (odds ratio (OR) = 2.13, 95% CI:1.50–3.04, *P* < 0.001), being a participant from the Cooperative Health FQHC (OR = 3.13, 95% CI:1.69–5.86, *P* < 0.001), having a smartphone (OR = 2.02, 95% CI:1.36–2.97, *P* < 0.001) and knowing what a fitness tracker is before taking the survey (OR = 1.79, 95% CI:1.29–2.49, *P* < 0.001). In the multivariate logistic regression model, participants who would like a fitness tracker were more likely to be among the 46–55 and 56–65 age groups and identified as non-Hispanic Black or African American. Participants who had a smartphone at the time of the survey and knew what a fitness tracker was before the survey were also more likely to want a fitness tracker. Not having a fitness tracker because they “are too expensive” and “do not understand how it can help participants, but want to learn” were also associated with answering yes to, “would you like to use a fitness tracker?” Not having a fitness tracker because “they are not helpful” or “do not want to commit to using it every day” were associated with answering “no.” These factors, including education and training on the value of these devices, could be considered when designing research studies and programs to improve digital health equity.

**Table 1:** Univariate and multivariate logistic regression to identify factors associated with “Would you like a fitness tracker?”.

Category	Would like a fitness tracker		Univariate OR (95% CI, *P* value)	Multivariate OR (95% CI, *P* value)
	No (*N* = 197) *N* (%)	Yes (*N* = 583) *N* (%)		
Center				
CHC	29 (14.7)	53 (9.1)	–	–
CHS	43 (21.8)	108 (18.5)	1.37 (0.77–2.44, *P* = 0.278)	2.02 (0.94–4.37, *P* = 0.073)
Coop	25 (12.7)	143 (24.5)	**3.13 (1.69–5.86,** ***P*** < **0.001)**	**2.15 (1.01–4.58,** ***P*** = **0.048)**
JH	39 (19.8)	117 (20.1)	1.64 (0.92–2.93, *P* = 0.094)	1.64 (0.66–4.06, *P*= 0.284)
SRH	13 (6.6)	37 (6.3)	1.56 (0.73–3.47, *P* = 0.264)	1.63 (0.55–4.97, *P* = 0.382)
SY	48 (24.4)	125 (21.4)	1.42 (0.81–2.49, *P* = 0.217)	1.12 (0.55–2.25, *P* = 0.759)
Age				
18–25	21 (10.7)	43 (7.4)	–	–
26–35	37 (18.8)	98 (16.8)	1.29 (0.67–2.45, *P* = 0.434)	1.62 (0.76–3.43, *P* = 0.207)
36–45	37 (18.8)	110 (18.9)	1.45 (0.76–2.75, *P* = 0.254)	1.96 (0.91–4.21, *P* = 0.085)
46–55	37 (18.8)	143 (24.5)	1.89 (0.99–3.55, *P* = 0.050)	**3.07 (1.42–6.64,** ***P*** = **0.004)**
56–65	38 (19.3)	123 (21.1)	1.58 (0.83–2.97, *P* = 0.158)	**2.96 (1.33–6.61,** ***P*** = **0.008)**
66–75	22 (11.2)	51 (8.7)	1.13 (0.55–2.34, *P* = 0.736)	1.88 (0.74–4.81, *P* = 0.185)
76+	5 (2.5)	15 (2.6)	1.47 (0.49–4.99, *P* = 0.511)	3.73 (0.90–16.71, *P* = 0.075)
Gender				
Cis man	73 (37.1)	131 (22.5)	–	–
Cis woman	112 (56.9)	429 (73.6)	**2.13 (1.50**–**3.04,** ***P*** < **0.001**)	1.49 (0.96–2.30, *P* = 0.072)
Gender minority	5 (2.5)	9 (1.5)	1.00 (0.33–3.37, *P* = 0.996)	0.90 (0.23–4.14, *P* = 0.888)
Prefer not to answer	7 (3.6)	14 (2.4)	1.11 (0.44–3.05, *P* = 0.823)	1.10 (0.33–4.11, *P* = 0.879)
Language				
English	137 (69.5)	387 (66.4)	–	–
Spanish	60 (30.5)	196 (33.6)	1.16 (0.82–1.65, *P* = 0.414)	1.37 (0.66–2.79, *P* = 0.393)
Education				
Less than high school	31 (16.4)	99 (17.3)	–	–
High school or some college	140 (74.1)	372 (65.1)	0.83 (0.53–1.29, *P* = 0.421)	0.61 (0.33–1.10, *P* = 0.108)
College degree or more	18 (9.5)	100 (17.5)	1.74 (0.92–3.36, *P* = 0.092)	1.20 (0.54–2.71, *P* = 0.662)
Race/ethnicity				
White	36 (18.3)	80 (13.7)	–	–
Black or African American	61 (31.0)	210 (36.0)	1.55 (0.95–2.51, *P* = 0.077)	**2.31 (1.01**–**5.34,** ***P*** = **0.048)**
Hispanic/Latinx/Spanish	76 (38.6)	229 (39.3)	1.36 (0.84–2.16, *P* = 0.205)	1.30 (0.56–3.05, *P* = 0.548)
AI/AN, Asian, ME/NA, or NH/PI	3 (1.5)	9 (1.5)	1.35 (0.38–6.35, *P* = 0.666)	1.38 (0.30–8.15, *P* = 0.695)
Multiple races or ethnicities	7 (3.6)	21 (3.6)	1.35 (0.55–3.68, *P* = 0.532)	1.51 (0.51–4.92, *P* = 0.473)
Other/prefer not to answer	14 (7.1)	34 (5.8)	1.09 (0.53–2.33, *P* = 0.813)	1.62 (0.63–4.40, *P* = 0.328)
Have a smartphone^a^	143 (73.3)	488 (84.7)	**2.02 (1.36**–**2.97,** ***P*** < **0.001)**	**2.66 (1.59**–**4.46,** ***P*** < **0.001)**
Knew what fitness tracker was before this survey^a^	81 (41.1)	323 (55.6)	**1.79 (1.29**–**2.49,** ***P*** < **0.001)**	**1.91 (1.25**–**2.93,** ***P*** = **0.003)**
Why do not have a fitness tracker^a^:				
They [Fitness Trackers] are not helpful	16 (8.5)	8 (1.4)	**0.15 (0.06**–**0.35,** ***P*** < **0.001)**	**0.15 (0.05**–**0.40,** ***P*** < **0.001)**
I do not know how to use it	30 (16.0)	109 (19.2)	1.25 (0.81–1.97, *P* = 0.327)	1.49 (0.87–2.60, *P*= 0.151)
They are too expensive	48 (25.5)	281 (49.4)	**2.85 (1.98**–**4.14,** ***P*** < **0.001)**	**2.71 (1.73**–**4.29,** ***P*** < **0.001)**
I do not understand how it can help me, but want to learn	12 (6.4)	89 (15.6)	**2.72 (1.51**–**5.34,** ***P*** = **0.002)**	**2.77 (1.43**–**5.79,** ***P*** = **0.004)**
I do not have access to the internet	10 (5.3)	23 (4.0)	0.75 (0.36–1.68, *P* = 0.459)	0.83 (0.36–2.05, *P* = 0.679)
I do not want to commit to using everyday	37 (19.7)	31 (5.4)	**0.24 (0.14**–**0.39,** ***P*** < **0.001)**	**0.30 (0.16**–**0.56,** ***P*** < **0.001)**

*OR* odds ratio, *CI* confidence interval, *CHC* Community Health Center, Inc., *CHS* Cherokee Health System, *Coop* Cooperative Health FQHC, *JH* Jackson Hinds Comprehensive Health Center, *SRH* Sun River Health, *SY* San Ysidro Health, *AI/AN* American Indian/Alaska Native, *ME/NA* Middle Eastern/North African, *NH/PI* Native Hawaiian/Pacific Islander.

A total of 780 patients from six Federally Qualified Health Centers responded to the question “Would you like a fitness tracker?” while 227 survey participants did not respond to the question. Participants answering yes agreed with one of the following statements: “Yes, to track my steps,” “Yes, to look at my heart rate,” or “Yes, to get more exercise”. The reference group answered, “No, I do not want one.” Bolded ORs reflect *P* values less than 0.05. The multivariate OR is mutually adjusted for all variables in the table.

^a^Reference rows omitted; reference group answered no.

Results from a qualitative content analysis were consistent with the quantitative findings. The top three themes were “no interest,” “lack of knowledge,” and “lost/broken device.” Over half of the qualitative responses to “why do not you have a fitness tracker” were coded as “No Interest” (52%, e.g.,: “I’ve never thought of having one,” “never considered it”). This may also be a result of limited awareness or knowledge of potential health impacts. Other common responses fell under the theme of lack of knowledge (18%, e.g.,: “didn’t know what they were”). While cost was not identified as a main theme among the open-ended questions, “lost/broken device” was prevalent, suggesting that cost may be a barrier to replacing a previously owned device. Our findings suggest that widespread adoption and use of digital health devices are possible across diverse communities, but would require a high-touch approach, including educational materials and public or private financial investment in devices. Limitations of the study include the surveyed patient sample may not be fully representative of the patient population of the six FQHCs, and the lack of a second parallel reviewer in the qualitative analysis.

The majority of patients surveyed are interested in using digital health devices and learning how these devices could improve health. However, cost and understanding how they work are important barriers that could prevent individuals from realizing the benefits of wearable digital health devices such as fitness trackers (Fig. 2). Consideration of cultural nuances are also important, for example with the terminology used to name these devices. In the course of this study, we learned that many Spanish-speaking participants were concerned that these devices could be used to track their movements, because of the word “trackers.” With the increase in telehealth and telemedicine use due to the COVID pandemic, access to digital health technologies is increasingly important. However, as the use of digital technology expands into health care, careful consideration is required to ensure that existing health equity gaps are not exacerbated and additional health equity gaps are not created.

While studies have been conducted on the use of wearables, very few have specifically sought input from UBR populations. In this study, patients were given the option to complete the survey in English or Spanish; one-third completed in Spanish. A Pew Research study^10^ found 21% of Americans use smartwatches or wearable fitness trackers. Use was greater for those with a higher annual household income and those identifying as white and/or non-Hispanic. More than 65% of the Pew survey participants identified as white and had an annual household income greater than $30 K per year. In contrast, over 70% of participants in our survey do not identify as white (36% identify as Black or African American compared to 10% in the Pew study, and 39% identify as Hispanic compared to 14% in the Pew study). Based on health center data, 90% of the patients at our recruitment centers have an annual income at or below 200% of the Federal Poverty Guideline. Data collected from FQHC *All of Us* participants indicate that 38.3% have an annual income of less than $10 K, 23.9% have incomes between $10 and $25 K, and 7.9% between $25 and $35 K, with 21.7% preferring not to answer. Our results align with recent findings by Tappen et al^11^, where significant differences in computer ownership, internet access, and use of digital health information were observed among older racial and ethnic minority individuals when compared to white adults of similar ages. Older age, lower education, lower-income, and minority racial and ethnicity identification predicted limited digital health information use^11^.

Wearables are evolving to monitor more specific health concerns, including diabetes and heart disease, two conditions that are prevalent in African American and Hispanic communities. Inclusive use of digital health technologies in research and clinical practice will likely require strategic planning for devices, infrastructure, and education about digital health technologies. Most individuals surveyed have smartphones and know how to install apps, but would benefit from additional information on how fitness trackers can be used to improve health. Since the cost of a device was one of the most hindering factors noted in the survey, investment is needed to help overcome this barrier to entry. There is a risk of increasing health disparities through noninclusion in research and clinical care using wearables and other digital health devices; the diverse participants in this study indicated interest in fitness trackers, but barriers such as cost and education exist. Future research to understand potential health disparities and inequity could investigate other evidence-based digital health solutions and real-world data beyond fitness trackers. The *All of Us* program is committed to engaging with diverse communities and building relationships with community leaders in order to gain trust, but is only one research program. The results of this survey suggest that additional investment in devices and educational materials from other clinical and research programs could contribute toward reducing disparities.

## Methods

### Study population

Between October - December 2020, we recruited a convenience sample of 1007 registered patients at six FQHCs: Cherokee Health Systems (TN), Community Health Center, Inc. (CT), Cooperative Health Center (SC), Jackson Hinds Comprehensive Health Center (MS), Sun River Health (NY), and San Ysidro Health (CA). Eligible participants were adult patients at one of the participating centers. Patients were reached by email, phone, by leaflets and in-person at the centers. Research study staff from each enrollment site read the consent statement to potential participants in their preferred language (Spanish or English), and verbal consent was obtained. Consent was obtained verbally and recorded in a password-protected file, housed on a secure server, at each site; this information was not shared. The Community Health Center, Inc. Institutional Review Board served as the IRB of record for all participating FQHCs (CHCI IRB# 1175, approved September 30, 2020). San Ysidro Health (SYH) obtained additional approvals from the SYH IRB (SYH Registration Project Number: RHP-R-092320-42).

### Data collection and survey questions

Surveys were administered by trained bilingual research staff. Participants received an invitation via email or received a QR code to access the survey independently. Surveys were also administered over the phone, where research staff from each center called participants, read the questions, and entered responses directly into SurveyMonkey. Some FQHCs handed out paper surveys and entered the responses into SurveyMonkey.

All FQHCs utilized the same questions with a personalized cover page for each recruitment center. The survey consisted of 27 questions designed to elicit a clear understanding of FQHC patient knowledge, use, and access to wearable fitness trackers. Survey participants were also asked about their willingness to share data with researchers and resources needed to facilitate sharing and adherence to fitness tracker use.

### Measures

The primary dependent variable was “would like a fitness tracker” based on the question “*Would you like a fitness tracker?*” Participants selected one of four options (“no,” “yes; to get more exercise,” “yes; to look at my heart rate,” or “yes; to track my steps”). These values were collapsed to create a binary outcome for “yes” (“yes; to get more exercise,” “yes; to look at my heart rate,” or “yes; to track my steps”) vs. “no.” We also asked if participants knew what a wearable device or fitness tracker was before taking this survey and if they ever previously owned or used a fitness tracker. Covariates were selected based on their relevance to the outcome variable and included: (i) socio-demographic characteristics (e.g., age, sex, gender, race, ethnicity, language, and educational level); (ii) knowledge and experience using electronic devices including the use of a smartphone and/or a fitness tracker; and (iii) perceptions, barriers and facilitators on the use of a fitness tracker (e.g., “Do you think these devices can give you helpful information about your health?”, “Would you recommend a fitness tracker to others?”, “Would you share the data with researchers?”, “Why do not you currently have a fitness tracker?”).

### Data analysis

We conducted a descriptive analysis of participant demographics and their association with two outcomes of interest: whether participants would like to use a fitness tracker and whether they had heard of a fitness tracker before taking the survey. Chi-squared tests of association were used to determine the extent to which survey responses differed by the following participant characteristics: center, categorical age (18–25, 26–35, 36–45, 46–55, 56–65, 66–75, 76 + ), race/ethnicity (White, Black or African American, Hispanic, Latino, or Spanish, American Indian/Alaska Native, Asian, Middle Eastern/North African, or Native Hawaiian/Pacific Islander I, Multiple races or ethnicities, Other/prefer not to answer), gender (cis man, cis woman, gender minority, prefer not to answer), education (less than high school, high school or some college, college degree or more), and language in which the survey was taken (Spanish, English) (Supplementary Table S1).

Univariate and multivariate logistic regression were additionally used to identify factors associated with “Would you like a fitness tracker?” (Table 1). The models included all variables from the descriptive analysis above along with: (i) whether participants knew what a fitness tracker was before taking the survey, (ii) owned a smartphone, and (iii) the following reasons for not currently owning a fitness tracker: “they are not helpful,” “I do not know how to use it,” “they are too expensive,” “I do not understand how it can help me but want to learn,” “I do not have access to the internet,” and “I do not want to commit to using it every day.” The multivariate model was mutually adjusted for all variables in the univariate models.

Ancillary qualitative content analysis was conducted on the open-ended question “Why do not you have a fitness tracker” to augment the quantitative findings. One hundred and eighty responses were iteratively coded by a single co-author until saturation was reached resulting in 12 themes.

All data analyses were conducted in R Studio Version 1.3.1056 running R-4.0.2^12,13^.

### Reporting summary

Further information on research design is available in the Nature Research Reporting Summary linked to this article.

## Supplementary information

Supplementary information

Reporting Summary

## Data Availability

The datasets generated during and/or analyzed during this study are available from the corresponding author on reasonable request.

## References

[1] Sim I (2019). Mobile devices and health. N. Engl. J. Med..

[2] Hirten, R. P. et al. Use of physiological data from a wearable device to identify SARS-CoV-2 infection and symptoms and predict COVID-19 diagnosis: observational study. *J. Med. Internet Res*. **23**, e26107 (2021). 10.2196/26107 PMC7901594 33529156

[3] Chandrasekaran, R. Katthula, V. & Moustakas, E. Patterns of use and key predictors for the use of wearable health care devices by US adults: insights from a national survey. *J. Med. Internet Res*. **22**, e22443 (2020). 10.2196/22443 PMC7600024 33064083

[4] Anderson, M. & Kumar, M. Digital divide persists even as lower-income Americans make gains in tech adoption. Pew Research Center, Washington, D.C. https://www.pewresearch.org/fact-tank/2019/05/07/digital-divide-persists-even-as-lower-income-americans-make-gains-in-tech-adoption/ (2019).

[5] Sieck, C. J. et al. Digital inclusion as a social determinant of health. *npj Digit Med*. **4**, 1–3 (2021). 10.1038/s41746-021-00413-8 PMC7969595 33731887

[6] All of Us Research Program Investigators (2019). The “All of Us” research program. N. Engl. J. Med..

[7] Mapes BM (2020). Diversity and inclusion for the *All of Us* research program: a scoping review. PLoS ONE.

[8] *All of Us* Research Program News and Events [Internet]. *All of Us* Research Program Expands Data Collection Efforts with Fitbit. https://allofus.nih.gov/news-events-and-media/announcements/all-us-research-program-expands-data-collection-efforts-fitbit. (2019).

[9] Holko, M. et al. Fitbit “Bring Your Own Device” data in the *All of Us* Research Program; AMIA Annual Meeting. https://knowledge.amia.org/72332-amia-1.4602255/t004-1.4605866/t004-1.4605867/3414532-1.4606075/3414532-1.4606076?qr=1 (2020).

[10] Vogels. About one-in-five Americans use a smart watch or fitness tracker. Pew Research Center, Washington, D.C. https://www.pewresearch.org/fact-tank/2020/01/09/about-one-in-five-americans-use-a-smart-watch-or-fitness-tracker/. (2020).

[11] Tappen RM (2021). Digital health information disparities in older adults: a mixed methods study. J. Racial Ethn. Health Disparities..

[12] RStudio Team. *RStudio: Integrated Development for R* (RStudio, PBC, Boston, MA, 2020).

[13] R Core Team. *R: A Language and environment for Statistical Computing* (R Foundation for Statistical Computing, Vienna, Austria, 2020).

