# Supplementary material for: Wearable fitness tracker use in federally qualified health center patients: strategies to improve the health of all of us using digital health devices
Source: NPJ Digit Med. 2022 Apr 25;5:53. doi: 10.1038/s41746-022-00593-x (PMC9038923; doi:10.1038/s41746-022-00593-x)
Supplement: Supplementary file 1 — Supplementary information [file 41746_2022_593_MOESM1_ESM.pdf]

Supplementary Information

Supplementary Table 1. Survey responses and associations by participant characteristics

	Would you like to own a fitness tracker?				Did you know what a wearable device or fitness tracker was before this survey?		
	**No**	**Yes**	**Total**		**No**	**Yes**	**Total**
**Center**							
**CHC**	29	53	82		44	79	123
**CHS**	43	108	151		85	111	196
**Coop**	25	143	168		91	157	248
**JH**	39	117	156		81	102	183
**SRH**	13	37	50		22	34	56
**SY**	48	125	173		89	108	197
**Total**	197	583	780		412	591	1003
$\chi2$=15.437 · df=5 · Cramer's V=0.141 · p=0.009					$\chi2$=6.034 · df=5 · Cramer's V=0.078 · p=0.303		
**Age**							
**18-25**	21	43	64		24	86	110
**26-35**	37	98	135		58	139	197
**36-45**	37	110	147		77	119	196
**46-55**	37	143	180		90	120	210
**56-65**	38	123	161		102	86	188
**66-75**	22	51	73		49	33	82
**76+**	5	15	20		12	8	20

**Total**	197	583	780		412	591	1003
χ2=5.529 · df=6 · Cramer's V=0.084 · p=0.478					χ2=56.679 · df=6 · Cramer's V=0.238 · p=0.000		
**Gender**							
**Cis man**	73	131	204		123	128	251
**Cis woman**	112	429	541		263	450	713
**Gender minority**	5	9	14		13	4	17
**Prefer not to answer**	7	14	21		13	9	22
**Total**	197	583	780		412	591	1003
χ2=19.458 · df=3 · Cramer's V=0.158 · Fisher's p=0.000					χ2=23.438 · df=3 · Cramer's V=0.153 · p=0.000		
**Language**							
**English**	137	387	524		254	440	694
**Spanish**	60	196	256		158	151	309
**Total**	197	583	780		412	591	1003
χ2=0.532 · df=1 · φ=0.029 · p=0.466					χ2=18.062 · df=1 · φ=0.136 · p=0.000		
**Education**							
**Did not attend**	1	3	4		2	3	5
**<9th grade**	30	96	126		91	43	134
**HS/GED**	108	255	363		195	243	438
**2 year college**	32	117	149		65	137	202

**4 year college**	17	99	116		43	154	197
**Graduate school**	1	1	2		0	2	2
**Total**	189	571	760		396	582	978
χ2=12.779 · df=5 · Cramer's V=0.130 · Fisher's p=0.011					χ2=80.394 · df=5 · Cramer's V=0.287 · Fisher's p=0.000		
Race/Ethnicity							
**AI/AN, Asian, ME/NA, or NH/PI**	3	9	12		4	9	13
**Black or African American**	61	210	271		143	216	359
**Hispanic/Latinx**	76	229	305		178	211	389
**Multiple races or ethnicities**	7	21	28		12	21	33
**Other/prefer not to answer**	14	34	48		25	32	57
**White**	36	80	116		50	102	152
**Total**	197	583	780		412	591	1003
χ2=3.544 · df=5 · Cramer's V=0.067 · Fisher's p=0.604					χ2=9.012 · df=5 · Cramer's V=0.095 · p=0.109		

Supplementary Table 1. Survey responses and associations by participant characteristics

Survey responses including chi-squared tests of association for the following participant characteristics: center, categorical age (18-25, 26-35, 36-45, 46-55, 56-65, 66-75, 76+), race/ethnicity (White, Black or African American, Hispanic/Latinx/Spanish, American Indian/Alaska Native (AI/AN), Asian, Middle Eastern/North African (ME/NA), or Native Hawaiian/Pacific Islander (NH/PI), Multiple races or ethnicities, Other/prefer not to answer), gender (cis man, cis woman, gender minority, prefer not to answer), education (less than high school, high school or some college, college degree or more), and language in which the survey was taken (Spanish, English).

Supplementary Figure 1: Map of Federally Qualified Health Centers

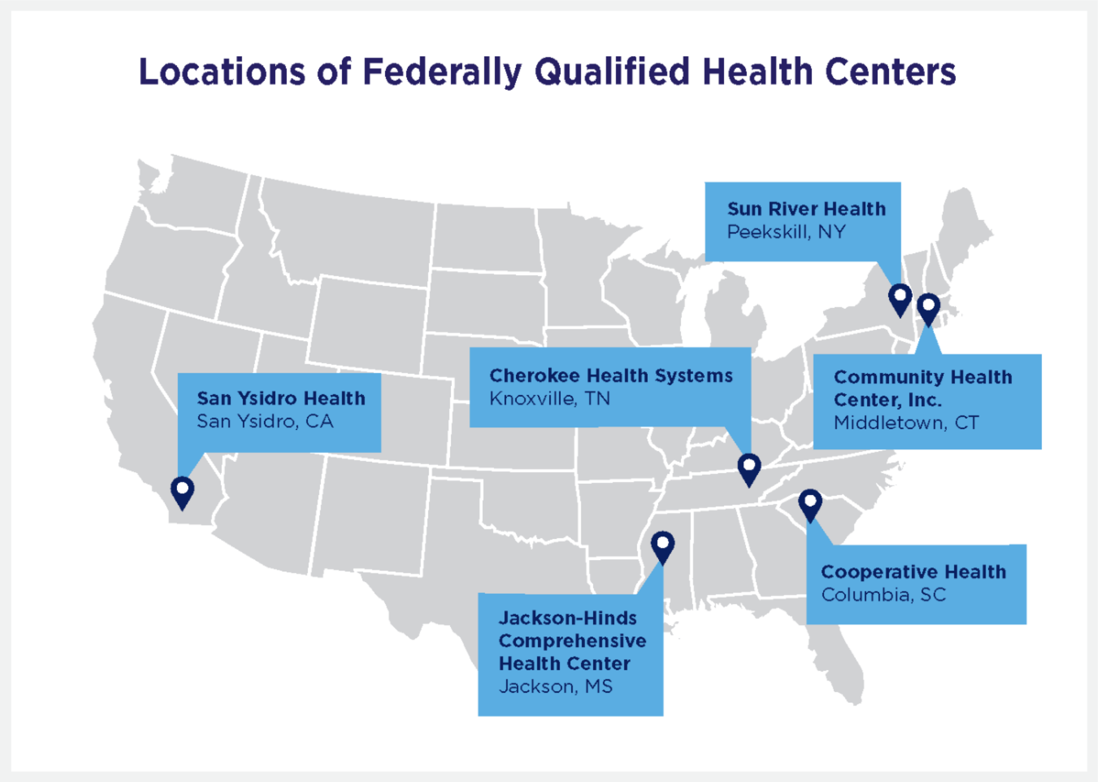

Supplementary Figure 1: Map of Federally Qualified Health Centers

Five Federally Qualified Health Centers (FQHCs) in the *All of Us* Research Program Consortium participated in the fitness tracker survey.
