# Supplementary material for: Wearable fitness tracker use in federally qualified health center patients: strategies to improve the health of all of us using digital health devices
Source: NPJ Digit Med. 2022 Apr 25;5:53. doi: 10.1038/s41746-022-00593-x (PMC9038923; doi:10.1038/s41746-022-00593-x)
Supplement: Supplementary file 2 — Reporting Summary [file 41746_2022_593_MOESM2_ESM.pdf]

nature portfolio

Corresponding author(s): Michelle Holko

Last updated by author(s): Feb 6, 2022

Reporting Summary

Nature Portfolio wishes to improve the reproducibility of the work that we publish. This form provides structure for consistency and transparency in reporting. For further information on Nature Portfolio policies, see our Editorial Policies and the Editorial Policy Checklist.

Statistics

For all statistical analyses, confirm that the following items are present in the figure legend, table legend, main text, or Methods section.

n/a	Confirmed	
☐	☒	The exact sample size (n) for each experimental group/condition, given as a discrete number and unit of measurement
☒	☐	A statement on whether measurements were taken from distinct samples or whether the same sample was measured repeatedly
☐	☒	The statistical test(s) used AND whether they are one- or two-sided<br>*Only common tests should be described solely by name; describe more complex techniques in the Methods section.*
☐	☒	A description of all covariates tested
☒	☐	A description of any assumptions or corrections, such as tests of normality and adjustment for multiple comparisons
☐	☒	A full description of the statistical parameters including central tendency (e.g. means) or other basic estimates (e.g. regression coefficient) AND variation (e.g. standard deviation) or associated estimates of uncertainty (e.g. confidence intervals)
☐	☒	For null hypothesis testing, the test statistic (e.g. F, t, r) with confidence intervals, effect sizes, degrees of freedom and P value noted<br>*Give P values as exact values whenever suitable.*
☒	☐	For Bayesian analysis, information on the choice of priors and Markov chain Monte Carlo settings
☒	☐	For hierarchical and complex designs, identification of the appropriate level for tests and full reporting of outcomes
☒	☐	Estimates of effect sizes (e.g. Cohen's d, Pearson's r), indicating how they were calculated

Our web collection on statistics for biologists contains articles on many of the points above.

Software and code

Policy information about availability of computer code

Data collection	Data were collected via SurveyMonkey, through the online portal.
Data analysis	All data analyses were conducted in R Studio Version 1.3.1056 running R-4.0.2 (R Studio Team 2020; R Core Team 2020). The "finalfit" package version 1.0.2 was used to conduct logistic regression analysis. Full code available to editors and reviewers upon request.

For manuscripts utilizing custom algorithms or software that are central to the research but not yet described in published literature, software must be made available to editors and reviewers. We strongly encourage code deposition in a community repository (e.g. GitHub). See the Nature Portfolio guidelines for submitting code & software for further information.

Data

Policy information about availability of data

All manuscripts must include a data availability statement. This statement should provide the following information, where applicable:

- Accession codes, unique identifiers, or web links for publicly available datasets
- A description of any restrictions on data availability
- For clinical datasets or third party data, please ensure that the statement adheres to our policy

The data that support the findings of this study are available from the senior author, YS upon reasonable request. The authors declare that data supporting the findings of this study are available within the paper and its supplementary information files. All data analyses were conducted in R Studio Version 1.3.1056 running R-4.0.2 (R Studio Team 2020; R Core Team 2020).

Field-specific reporting

Please select the one below that is the best fit for your research. If you are not sure, read the appropriate sections before making your selection.

☐ Life sciences ☒ Behavioural & social sciences ☐ Ecological, evolutionary & environmental sciences

For a reference copy of the document with all sections, see nature.com/documents/nr-reporting-summary-flat.pdf

Behavioural & social sciences study design

All studies must disclose on these points even when the disclosure is negative.

Study description	A quantitative study was conducted to understand fitness tracker use among Federally Qualified Health Center patients.
Research sample	We surveyed 1,007 adult patients at six Federally Qualified Health Centers regarding fitness tracker access and use. These FQHCs were selected because of their participation in the All of Us Research Program (AoURP) and their patient population are representative of populations underrepresented in biomedical research (UBR).
Sampling strategy	Purposive sampling was employed to reach patients of lower socioeconomic status, typical of FQHCs and who fall into at least one UBR category (race, ethnicity, gender, age, educational attainment). No sample size calculation was performed, however targets were based on the sample size of patients previously enrolled for the AoURP.
Data collection	Patients from each FQHC received an email or phone call explaining the study. If the patient agree to participate they used the link provided in the email to complete the survey on SurveyMonkey. If the patient agree to participate over the phone, the research team read the survey questions and entered the participant's response into SurveyMonkey. One center handed out paper copies of the survey for patients to complete independently or in the presence of the research team member. Responses from paper survey were then entered manually into SurveyMonkey. Another center handed out a brief introduction of the study with a QR code. Patients used the QR code to access the survey independently. No PHI or other identifiable information were collected; all surveys were anonymous. The study received a waiver of written consent and was approved by the Community Health Center, Inc. Institutional Review Board. The researchers were not blinded to the study hypothesis during data collection.
Timing	The study received IRB approval in early October 2020 and data collection began immediately and continued to the end of December 2020.
Data exclusions	No data were excluded from the analysis.
Non-participation	The declination rate was 70%; no participants dropped out.
Randomization	Participants were not allocated to experimental groups.

Reporting for specific materials, systems and methods

We require information from authors about some types of materials, experimental systems and methods used in many studies. Here, indicate whether each material, system or method listed is relevant to your study. If you are not sure if a list item applies to your research, read the appropriate section before selecting a response.

Materials & experimental systems

n/a	Involved in the study	
☒	☐	Antibodies
☒	☐	Eukaryotic cell lines
☒	☐	Palaeontology and archaeology
☒	☐	Animals and other organisms
☐	☒	Human research participants
☒	☐	Clinical data
☒	☐	Dual use research of concern

Methods

n/a	Involved in the study	
☒	☐	ChIP-seq
☒	☐	Flow cytometry
☒	☐	MRI-based neuroimaging

Human research participants

Policy information about studies involving human research participants

Population characteristics	See above.

Recruitment	Active patients of the participating FQHCs were approached by phone, email or in person and invited to complete the survey. Researchers obtained a waiver of documentation of informed consent, as no PHI was collected with the survey.
Ethics oversight	The Community Health Center, Inc. Institutional Review Board approved this study; study ID #1175.

Note that full information on the approval of the study protocol must also be provided in the manuscript.
